# Epigallocatechin-3-gallate inhibits the collagen accumulation of oral submucous fibrosis induced by arecoline

**DOI:** 10.3389/fphar.2025.1540559

**Published:** 2025-01-31

**Authors:** Ge Gao, Caipeng Lin, Ruibo Li, Xi Xie, Hai-Bin Luo

**Affiliations:** ^1^ Key Laboratory of Tropical Biological Resources of Ministry of Education and Hainan Engineering Research Center for Drug Screening and Evaluation, School of Pharmaceutical Sciences, Hainan University, Haikou, China; ^2^ School of Life and Health Sciences, Hainan University, Haikou, China; ^3^ Song Li’s Academician Workstation of Hainan University (School of Pharmaceutical Sciences), Sanya, China

**Keywords:** epigallocatechin-3-gallate, green tea, oral submucous fibrosis, arecoline, extracellular matrix

## Abstract

**Objective:**

Oral submucous fibrosis (OSF) is a chronic oral mucosal disease, which exerts a profound impact on patients’ daily life and currently lacks efficacious therapeutic interventions. Epigallocatechin-3-gallate (EGCG), the abundant polyphenol found in green tea, exhibits remarkable anti-fibrotic effects on the skin. However, the research on OSF regarding EGCG is relatively limited.

**Purpose:**

We aimed to investigate the potential therapeutic effect of EGCG against OSF using an arecoline (ARE) -induced rat model and primary rat oral fibroblasts.

**Methods:**

Primary rat oral mucosal fibroblasts (ROMF) were isolated and identified. Optimal ARE concentrations were established using the Cell Counting Kit-8. The impact of ARE on extracellular matrix (ECM)-related protein expression was assessed through RT-qPCR and Western blot techniques. Similarly, the effects of EGCG on ARE-induced ECM changes in ROMF were evaluated. The study also established an OSF model in Sprague-Dawley rats, induced by ARE, with pathological changes characterized using HE and Masson’s staining, further assessing the impact of ARE on ECM-related protein expression in rat oral tissues through RT-qPCR and Western blot methods.

**Results:**

EGCG effectively suppressed the ARE-induced ECM components while concurrently improving the OSF pathological process *in vitro* and *in vivo*.

**Conclusion:**

The results indicate that the natural product EGCG effectively suppressed the increased ECM components induced by ARE and concurrently improved the OSF pathological process, indicating that EGCG could be potentially a novel anti-fibrotic candidate drug for the treatment of OSF.

## 1 Introduction

Oral submucous fibrosis (OSF) is defined as one of the chronic progressive oral diseases, that produces scars, tissue fibrosis, and even leads to squamous cell carcinoma (Li et al., 2019). It affects more than 5 million people and is prevalent in South Asia and among Pacific Islanders (Muller and Tilakaratne, 2022). Long-term chewing of areca nuts plays an important role in the progression of OSF, affecting over 10% of the population (Warnakulasuriya and Chen, 2022; Liu et al., 2015). The reason is that areca nuts promote the proliferation of fibroblasts and the deposition of collagen (Liu et al., 2022). Fibroblasts are critical to both the structural integrity and repair mechanisms of tissues, which positions them as pivotal players in fibrosis research. These cells are the primary architects of the extracellular matrix (ECM), which undergoes significant deposition and remodeling in fibrotic conditions. When exposed to fibrogenic factors, fibroblasts transform into myofibroblasts, markedly increasing their ECM production. This transition and the resultant ECM buildup are definitive characteristics of fibrotic diseases, underscoring the relevance of fibroblasts in modeling fibrosis. Fibronectin (FN) and Collagen I are ECM components critical in fibrosis, with FN initiating and Collagen I enhancing tissue stiffening. α-SMA, marking myofibroblast activation, drives ECM contraction. Together, these proteins contribute to the pathological remodeling and stiffening in fibrotic diseases.

In 2012, the International Agency for Research on Cancer and the World Health Organization have categorized areca nut/betel nut as category I carcinogens to humans. Areca nut is the seed of the *Areca catechu L* which is widely distributed in tropical and subtropical districts, such as South and Southeast Asia (Wang et al., 2024). The main ingredients in areca nut include crude fiber, fat, carbohydrates, polyphenols, tannin acid, and alkaloids (Yuan et al., 2024; Oliveira et al., 2021). The main alkaloids of areca nut involve arecoline, arecaidine, guvacine, and guvacoline (Chuang et al., 2022). Our research group previously demonstrated through network pharmacology studies that arecoline, the primary component of areca nut, is highly associated with the incidence of OSF (Li et al., 2024). The primary treatments for OSF include drug therapy, surgery, and physiotherapy, with drug therapy being the most common. Corticosteroids (Al-Maweri et al., 2023) [e.g., prednisolone (Al-Maweri et al., 2023)], triamcinolone (Chen et al., 2023) provide rapid relief by reducing inflammation and pain, especially in early OSF, but long-term use has side effects like weight gain and infections without stopping disease progression. Anti-fibrosis agents: collagenase helps break down fibrotic tissue and improves mouth opening but requires invasive injections and is limited in availability. Vasodilators (e.g., Pentoxifylline) enhances microcirculation, potentially improving blood flow in OSF patients (Chen et al., 2023; Rai et al., 2023). Antioxidants: Vitamin E (Shah et al., 2023; Mahdani et al., 2024) may improve oral flexibility but shows slow and inconsistent results. Topical capsaicin provides temporary pain relief but can irritate. Other drugs: Hyaluronic acid (Guo et al., 2023) and L-arginine (Shieh et al., 2009) may improve tissue repair and flexibility, though the evidence is limited and side effects like discomfort may occur. Currently, the development of therapeutic agents to hold back Oral submucous fibrosis has not been successful. Therefore, there is still a need to explore and develop therapeutic strategies to treat Oral submucous fibrosis.

Green tea is one of the oldest and the most widely traditional beverages (especially in China) in history, with the connotation of tea polyphenols. Epigallocatechin-3-gallate (EGCG) is the biologically active constituent responsible for the therapeutic action of tea polyphenol and is proven that the EGCG acts on multiple benefits (Luo et al., 2024). Compared to other polyphenols, EGCG is widely used as an anti-inflammatory agent, antibiotic, and antioxidant (Xu et al., 2021; Dassanayake et al., 2021; Surma et al., 2023). Several studies indicate the preventive effects of EGCG against fibrosis at various sites, including the skin (Hu et al., 2023; Song et al., 2023), lung (Cohen et al., 2024; Tanaka et al., 2022), breast (Piwowarczyk et al., 2020), colon (Chen et al., 2015), liver (Mostafa-Hedeab et al., 2022; George et al., 2022), and prostate (Zhang et al., 2023). As aforementioned, EGCG is an excellent antifibrotic agent, but there are relatively limited studies on the inhibitory effect of EGCG on ARE-induced OSF. Hence, this study was conducted to investigate the antifibrotic effects of EGCG on ARE-induced rat oral mucosal fibroblasts (ROMF) and rat models.

## 2 Materials and methods

### 2.1 Isolation of primary rat oral mucosal fibroblasts

The healthy SD rat was anesthetized and euthanized using an approved method with isoflurane. The buccal mucosa was aseptically excised from the oral cavity. Small pieces of the mucosa were dissected and individually placed on culture dishes. Primary fibroblasts were then cultured at 37°C in a humidified incubator with 5% CO₂.

### 2.2 Cell culture proliferation and differentiation

Fibroblast cells were cultivated in a mixture of DMEM/F12 medium (Gibco; 8,120,254) enriched with 10% fetal bovine serum (NEWZERUM; FBS-PA500), along with antibiotics-100 μg/mL streptomycin and 100 U/mL penicillin (Beyotime; C0222). Once the cells reached a density of about 80%, they were removed from the culture vessel by treatment with a 0.25% trypsin-EDTA solution (Gibco; 25,200,056) for 3 minutes at a temperature of 37°C. The cell suspension was then neutralized with the full-strength growth medium to inhibit the trypsin’s action.

### 2.3 Cell immunostaining

ROMF cells were fixed with 4% paraformaldehyde for 15 min at room temperature, followed by permeabilization with 0.1% Triton X-100 for 10 min. Cells were then blocked with 5% normal goat serum (Solarbio; SL038) for 60 min to prevent non-specific binding. After blocking, cells were incubated overnight at 4°C with primary antibodies against Vimentin (Beyotime; AF0318), Keratin17 (CST; 12,509) and α-SMA (Abcam; ab7817) diluted in 1% blocking buffer. The following day, fibroblasts were incubated for 1 h at room temperature in the dark with Cy3-conjugated (Boster; BA1032) and DyLight 488-conjugated secondary antibodies (Boster; BA1126). Nuclei were counterstained with DAPI (Solarbio; C0065) for 10 min, followed by PBS washes. Cells were then mounted with a suitable mounting medium for imaging. Each step was followed by extensive washing with PBS. Three final washes were conducted to remove any residual reagents. The stained cells were examined and imaged using a fluorescence microscope.

### 2.4 Cell viability assay

ROMF cells were dispensed into 96-well microplates with an initial concentration of 5,000 cells per well. Following the application of the specified experimental treatment, the Cell Counting Kit-8 (CCK8)reagent (Beyotime; C0038) was introduced into the wells in accordance with the supplier’s guidelines. The plates were subsequently maintained at a temperature of 37°C for a period of 60 min to facilitate the colorimetric reaction. The quantification of cellular viability was determined by spectrophotometric analysis at an absorbance wavelength of 450 nm, employing a microplate spectrophotometer.

### 2.5 ROMF models and treatment

After achieving 80% confluence, ROMF were seeded in a six-well plate using a 2% low-serum medium at a density of 1.8 × 10^5^ cells per well and incubated overnight. Subsequently, the medium was replaced with a standard culture medium supplemented with EGCG at final concentrations of 10, 30, and 90 µM. 2 h following the addition of EGCG, 128 µM of ARE was administered. ROMF were then incubated for an additional 48 h before being washed twice with PBS at 4°C.

### 2.6 Animals

Adult male Sprague Dawley rats were sourced from Biotechnology Co., Ltd. Beijing, China. Animals were housed on a standard 12:12 h light-dark cycle with free access to food and water. All the animal experiments were conducted with the use of the protocols (protocol No.2023001) approved by the Institutional Animal Care and Use Committee of Hainan University.

### 2.7 Animal models and treatment

Rats were divided into three groups, each consisting of six rats, based on mouth opening and body weight to ensure no statistically significant differences were present at the start of the experiment. The groups were classified as follows: normal, ARE (18 mg/mL)-induced model group, and ARE + EGCG-treated group (EGCG dose: 100 mg/kg body weight). Both the ARE and ARE + EGCG groups were subjected to an arecoline-induced model; every two days for 20 weeks, the buccal mucosa was rubbed 20 times on each side using an eyelash brush exerting a consistent force of 6 N to simulate the pathological condition. After each modeling session, the rats were deprived of both food and water for a period of 2 h. The ARE + EGCG group received EGCG daily by intragastric gavage at a dose of 100 mg/kg body weight for the same period. At the end of the 20-week experiment, the rats were euthanized, and oral tissues were collected and stored at −80°C for subsequent analyses.

### 2.8 Hematoxylin-Eosin (HE) staining

Oral tissues were preserved in 4% paraformaldehyde and embedded in paraffin sections (5 μm in thickness) and stained with Hematoxylin and Eosin (H&E). The stained sections were examined under a light microscope to assess oral tissue damage and morphological changes.

### 2.9 Masson staining

Dewaxed and rehydrated paraffin sections were first stained with Regaud’s hematoxylin solution to visualize cell nuclei. Following this, the sections were treated with an acidic dye solution to stain collagen fibers. The Masson staining procedure allowed for the differentiation of collagen deposition from other tissue components. Finally, the stained sections were observed under a light microscope to assess collagen content and distribution within the oral tissues.

### 2.10 Quantitative reverse transcription polymerase chain reaction

Total RNA was purified from ROMF and oral mucosa samples utilizing RNAiso Plus (Takara; 9109), following the protocol provided by the manufacturer. The RNA obtained was then converted into complementary DNA (cDNA) with the aid of the ABScript III RT Master Mix for qPCR with gDNA Remover (Abclonal; RK20429). The subsequent reverse transcription-quantitative polymerase chain reaction (RT-qPCR) was executed using TB Green^®^ Premix Ex Taq™ II (Tli RNaseH Plus) (Takara; RR820A) on a LightCycler^®^ 480 Instrument I system (Roche). The thermal cycling profile consisted of an initial denaturation step at 95°C for 30 s, succeeded by 40 cycles of denaturation at 95°C for 5 s and annealing/extension at 60°C for 30 s. Relative gene expression was calculated employing the 2^^−ΔΔCT^ method, with normalization to an endogenous control gene’s expression. The oligonucleotide primers employed in this research were fabricated by Sangon Biotech (Shanghai, China), and their sequences are detailed in Table 1.

**TABLE 1:** Primer sequences used for RT-qPCR.

Gene	Sense primer (5′-3 ′)	Antisense primer (5′-3 ′)
*Fn1*	CCA​CCA​TCA​CTG​GTC​TGG​AG	GGG​TGT​GGA​AGG​GTA​ACC​AG
*Col1a*	TCACCTACAGCACGCTTG	GGTCTGTTTCCAGGGTTG
*Actb*	CTG​AGA​GGG​AAA​TCG​TGC​GT	AGG​GAG​GAA​GAG​GAT​GCG​G

### 2.11 Western blot

Proteins extracted from ROMF and oral mucosa were disrupted and solubilized in RIPA lysis buffer (Beyotime; P0013B), which was enriched with a Protease Inhibitor Cocktail (Beyotime; P1045). The total protein concentration was measured using the BCA Protein Assay Kit (Thermo Fisher; 23,227). The samples were separated by SDS-polyacrylamide gel electrophoresis (PAGE) using 8% acrylamide gels and then transferred to PVDF membranes. After incubation, primary antibodies were incubated overnight at 4°C and secondary antibodies were incubated. The following antibodies were used. FN (Proteintech; 66042-1-Ig; 1:1000), Collagen I (NSJ Bioreagents; R32436; 1:1,000), Alpha-smooth muscle actin (α-SMA) (Servicebio; GB111364-100; 1:1,000), and β-Tubulin (Servicebio; GB111364-100; 1:1,000). Horseradish peroxidase (HRP)-linked secondary antibodies (Beyotime; A0350; 1:200). All protein bands were visualized using an enhanced chemiluminescence (ECL) detection system.

### 2.12 Statistical analysis

The results are expressed as mean ± standard deviation (SD). For statistical processing, GraphPad Prism version 9.0 software was utilized. One-way analysis of variance (ANOVA) was used to evaluate differences among groups, followed by Tukey’s multiple comparison test for *post hoc* analysis. Statistical significance was set at *p* < 0.05.

## 3 Results

### 3.1 Isolation and identification of primary cultured rat oral fibroblasts

**FIGURE 1:**
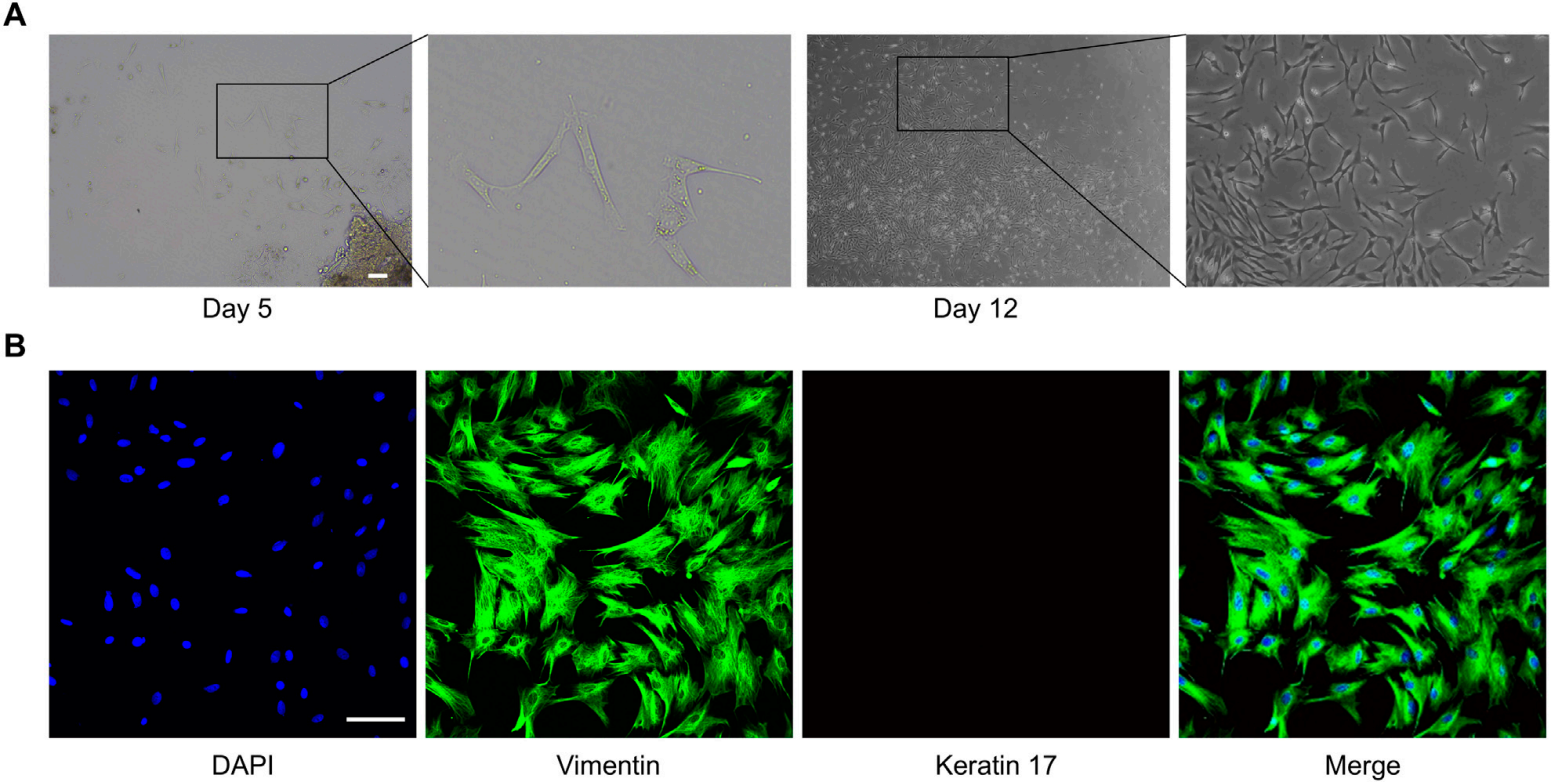
Extraction and characterization of primary fibroblasts from rat oral mucosa. **(A)** One of the representative results of cell migration from the rat oral cavity tissue pieces, the scale bar = 200 µm. **(B)** One of the representative results of immunofluorescence staining of Vimentin (green) and Keratin 17 (red), the scale bar = 100 µm.

Oral fibroblasts were cultured from oral cavity tissues by the explant technique. Initially, the majority of oral fibroblasts displayed a long shuttle-shaped morphology on days 5 and 12 of culture (Figure 1A). Immunofluorescence microscopy confirmed that the fibroblasts were negative for keratin 17 (red), typically a marker of epithelial cells, but were positive for vimentin (green), which reaffirms their identity as fibroblasts (Figure 1B). ROMF were serially passaged and used for experiments until passage 6.

### 3.2 Effects of ARE-induced activity of ROMF

**FIGURE 2:**
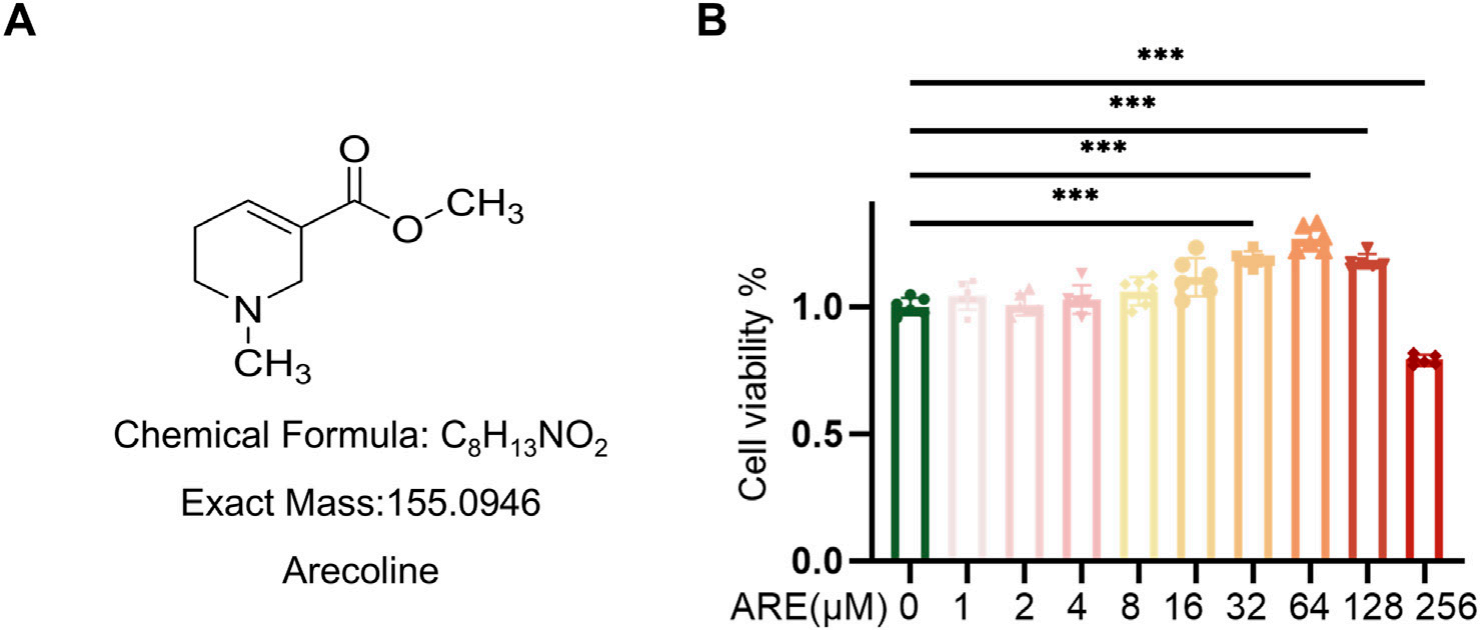
CCK-8 assays evaluated the toxicity of ARE in ROMF. **(A)** The structural formula of arecoline. **(B)** Cell counting kit-8 (CCK8) assay showing proliferative capacity on ROMF (n = 3). **p < 0.01, ***p < 0.001. Data are represented as Mean ± SD.

To identify ARE (Figure 2A) capable of exerting toxicity in fibroblasts, we assessed ARE cytotoxic effect at gradient concentrations ranging from 0 to 256 μM for 48 h. Cell viability was determined by CCK8 assay according to the manufacturer’s instructions. The fibroblast proliferation abilities of the 32, 64 and 128 μmol/L ARE groups were significantly improved compared with a non-ARE group (Figure 2B). In the 256 μM ARE group, the proliferation ability of fibroblasts was significantly inhibited. Hence, we selected 32, 64 and 128 μmol/L for a period of 48 h treatment for further studies.

### 3.3 ARE promoted abnormal growth of ROMF

**FIGURE 3:**
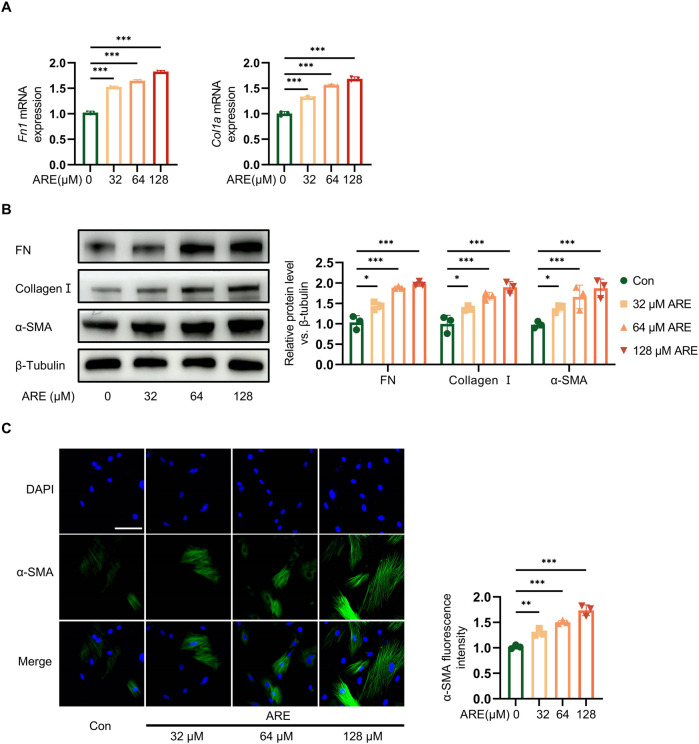
Effects of ARE promoted abnormal growth of OSF. **(A)** Real-time polymerase chain reaction (RT-qPCR) quantification of Fn and collagen Ⅰ mRNA levels (fold change normalized to Controls) in ROMF (n = 3). **(B)** Representative Western blots and quantification of FN, Collagen Ⅰ and α-SMA protein levels in ROMF (fold change normalized to Controls) (n = 3). **(C)** Immunofluorescence staining to detect the α-SMA (green) expression levels in ROMF, blue (DAPI staining) represents nuclei (n = 3), the scale bar = 100 µm *p < 0.05; **p < 0.01; ***p < 0.001. Data are represented as Mean ± SD.

Subsequent experiments were executed to explore whether ARE influenced the fibrosis progression. Fibroblasts were pretreated with different concentrations of ARE (32, 64 and 128 μmol/L) for 48 h. The expression level of fibroblast activation markers FN, Collagen І and α-SMA, protein in fibroblasts cultured with gradient concentrations of ARE were analyzed (Figure 3A).

Immunofluorescence results also showed that fibroblasts expressed ɑ-SMA (green) were the highest at 128 μmol/L of ARE. RT-qPCR result showed the high mRNA expressions of FN and Collagen I. Meanwhile, Western blot analysis showed that ARE significantly enhanced the levels of fibrosis marker proteins ɑ-SMA, Collagen I and FN. They showed significantly increased in a dose-dependent manner upon ARE stimulation (32, 64 and 128 μmol/L) (Figures 3B, C).

These findings demonstrate that ARE induces fibrosis in oral fibroblasts *in vitro*, suggesting a pivotal role in the progression of the disease. The most pronounced effects were observed at a concentration of 128 μmol/L of ARE over 48 h. This specific concentration was chosen to develop an oral submucous fibrosis (OSF) cell model.

### 3.4 EGCG alleviated fibrosis in ARE-induced fibroblasts

**FIGURE 4:**
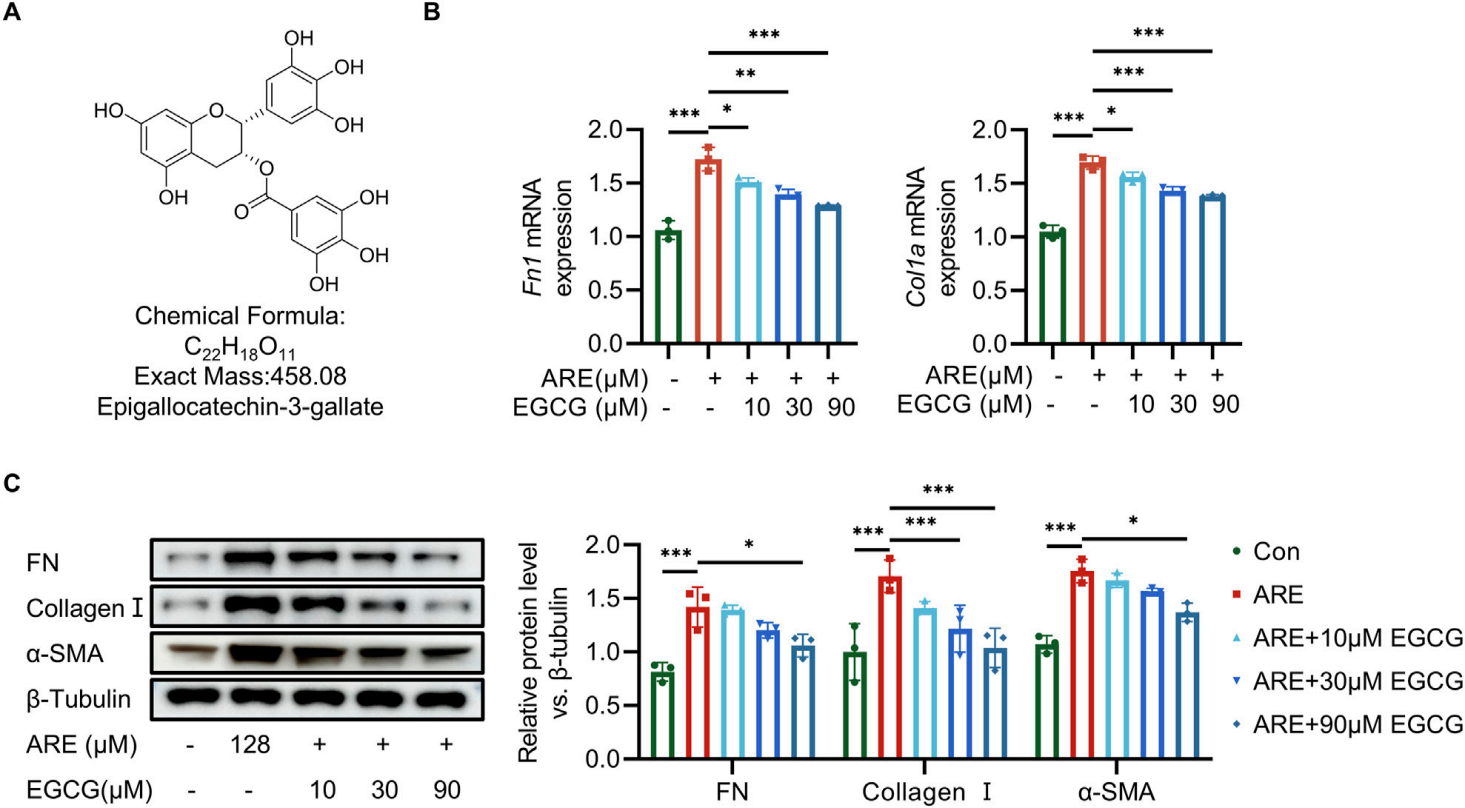
EGCG exerts oral-protective effects on ARE-induced oral submucous fibrosis. **(A)** The structural formula of EGCG. **(B)** RT-qPCR quantification of Fn and collagen Ⅰ mRNA levels (fold change normalized to controls) in ROMF (n = 3). **(C)** Representative Western blots and quantification of FN, Collagen Ⅰ and α-SMA protein levels in ROMF (fold change normalized to Controls) (n = 3). *p < 0.05; **p < 0.01; ***p < 0.001. Data are represented as Mean ± SD.

We then further tested the protective effects of EGCG (Figure 4A) preconditioning against ARE at 128 μmol/L concentrations. Fibroblasts were exposed to 128 μmol/L ARE for 48 h, and were treated with different concentrations of EGCG (10, 30 and 90 µM). The expression levels of FN and Collagen I were detected using RT-qPCR. Our results revealed that the EGCG could impede the increase of the mRNA level of FN and Collagen I compared to the ARE group (Figure 4B). As illustrated in Figure 4C, compared to the ARE group, EGCG treatment significantly attenuated FN, Collagen I, and α-SMA accumulation in the ROMF. This finding was consistent with the results of Western blot experiments.

### 3.5 EGCG reduced oral fibrosis and condensed collagen-deposition areas in OSF rats induced by ARE

**FIGURE 5:**
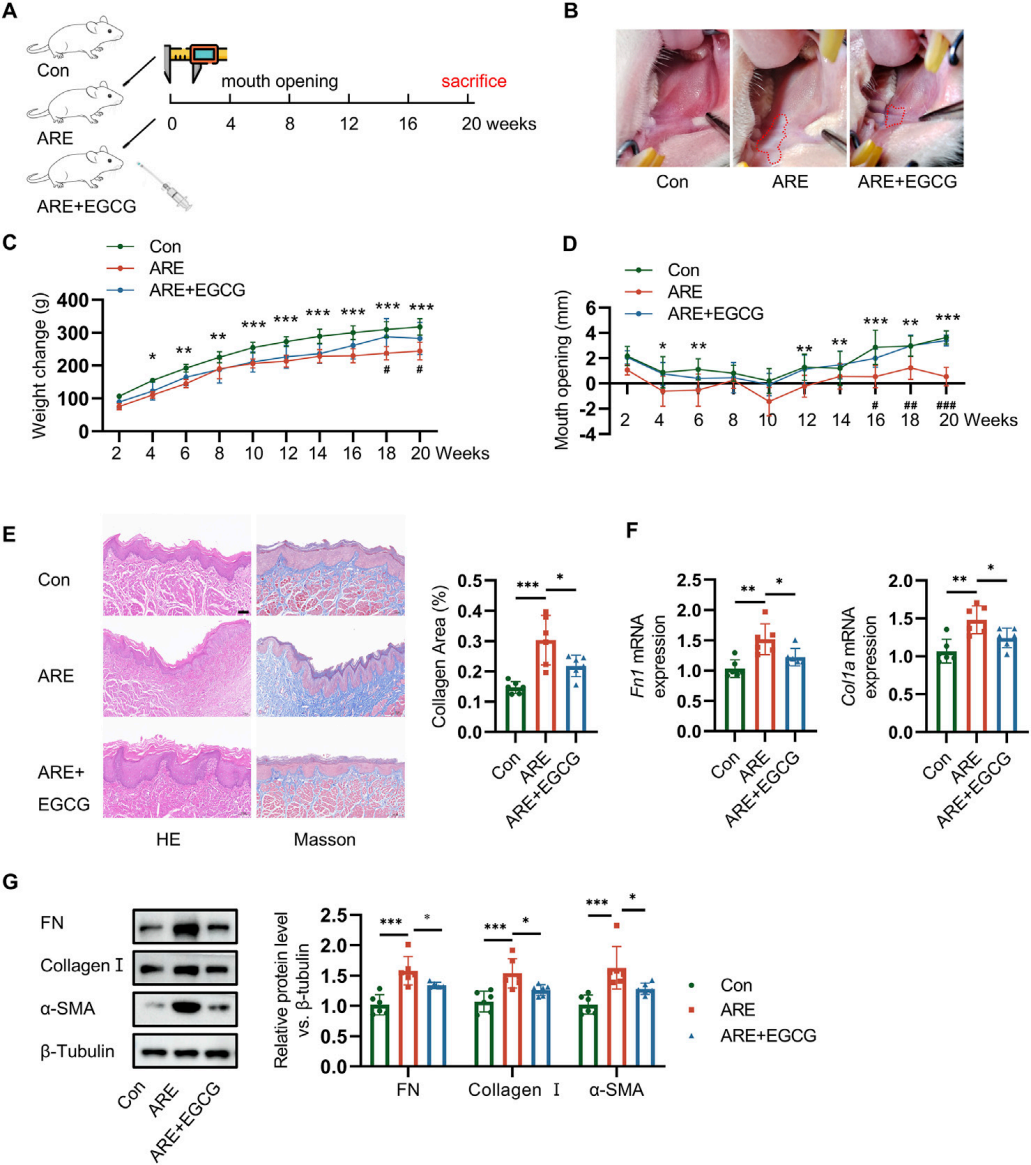
EGCG reduced fibrotic and condensed collagen-deposition areas in rats with ARE. **(A)** Experimental timeline of arecoline-induced oral submucous fibrosis. **(B)** Changes of oral mucosa in rats. **(C)** Changes of weight in rats. **(D)** Changes of mouth opening in rats. **(E)** One of the representative results of HE and Masson staining of the OSF tissue samples, the scale bar = 100 µm. **(F)** RT-qPCR quantification of Fn and collagen Ⅰ mRNA levels (fold change normalized to controls) in rats (n = 6). **(G)** Representative Western blots and quantification of FN, Collagen Ⅰ and α-SMA protein levels in rats (fold change normalized to Controls) (n = 6). *p < 0.05; **p < 0.01; ***p < 0.001 vs. the Control group. #*p* < 0.05; ##*p* < 0.01; ###*p* < 0.001 vs. the ARE group. Data are represented as Mean ± SD.

ARE was used to stimulate the oral mucosa of rats, inducing pathological changes that resemble those seen in human OSF. In this study, a rat model of OSF was established by applying ARE to the oral mucosa using an eye brush, with 20 brush strokes on each side, over 20 weeks (Li et al., 2019; Gao et al., 2018) (Figure 5A). The lesion areas in the EGCG treatment group were notably smaller compared to those in the ARE group (Figure 5B). Quantitative analysis of weight change and mouth opening revealed a reduction in disease after EGCG treatment compared with the OSF group (Figures 5C,D). To further investigate whether EGCG attenuates fibrosis and collagen deposition in OSF rats, we performed Hematoxylin-Eosin (HE) and Masson’s trichrome staining (Figure 5E). HE staining revealed epithelial atrophy, flattening of the epithelial layer, infiltration of submucosal inflammatory cells, and dense collagen in the lamina propria of OSF tissues. Masson staining further confirmed the widespread deposition of blue-purple collagen fibers, indicating increased fibrosis. Additionally, to corroborate the protective effects of EGCG on ARE-induced oral fibrosis, we assessed the expression levels of FN and collagen I via RT-qPCR. Our results demonstrated that EGCG treatment significantly reduced the mRNA level of FN and Collagen I in comparison to the OSF group (Figure 5F). As shown in Figure 5G, EGCG treatment also reduced Collagen deposition in the oral mucosa of rats with fibrosis.

## 4 Discussion

OSF is a progressive disease characterized by increasing tightness of the cheeks and mouth, significantly impacting patients’ quality of life. The condition is driven by extracellular matrix (ECM) deposition due to an imbalance in collagen metabolism. Key ECM components, such as Collagen I and FN, are critical drivers of the fibrotic process in OSF (Patrawalla et al., 2023; Nashchekina et al., 2022). Oral fibroblasts, responsible for synthesizing and remodeling the ECM, play a pivotal role in maintaining tissue homeostasis. However, in OSF, these fibroblasts become abnormally activated, leading to excessive production of ECM components like Collagen I and FN, while ECM degradation is impaired, leading to collagen accumulation, tissue stiffening, loss of elasticity, and characteristic fibrosis. The imbalance between collagen synthesis and degradation contributes to the progression of OSF, manifesting as restricted mouth opening (trismus), functional impairment, and a heightened risk of malignant transformation (Kokash et al., 2023).

Understanding the mechanisms behind this imbalance offers valuable insights into OSF pathophysiology and highlights potential therapeutic targets, such as modulating TGF-β signaling or enhancing matrix metalloproteinase activity, to restore normal collagen metabolism and prevent further tissue damage (Liu et al., 2021). However, to date, there is no satisfactory treatment for OSF. Tea drinking, a long-standing tradition in China, has been extensively studied for its anti-inflammatory (Xu et al., 2024; Wang et al., 2021; Niu et al., 2022) and antioxidant properties (Xing et al., 2019; Yang et al., 2023), primarily attributed to tea polyphenols. Based on the health benefits of tea, we aimed to investigate the effects of EGCG, on oral fibrosis. In our study, we established an OSF rats model using a combination of mascara brush friction and ARE application, simulating the effects of betel nut chewing. This model effectively mimics the early stages of human oral fibrosis, though the experimental timeline remains shorter than real-world disease progression. The results demonstrated that intragastric administration of EGCG for 20 weeks significantly alleviated oral pathological changes, improving buccal mucosa damage, increasing mouth opening, reducing ECM deposition, and decreasing fibrosis in OSF rats. Despite these promising findings, our study has several limitations, including a small sample size, short model duration, and relatively mild pathological changes. Consequently, our model does not fully capture the chronic nature of human oral fibrosis. Future research should involve longer observation periods and deeper exploration into the mechanisms by which EGCG mitigates oral fibrosis.

## 5 Conclusion

Our results indicated that the natural product EGCG alleviated oral fibrosis in ARE-induced OSF. The oral protective effects of EGCG may contribute to inhibiting collagen deposition. Our study provides a new promising treatment approach for OSF.

## Data Availability

The original contributions presented in the study are included in the article/supplementary material, further inquiries can be directed to the corresponding authors.
